# Anxiety Regarding COVID-19 Is Related to Attentional Control: The Mediating Role of Anxiety Sensitivity

**DOI:** 10.3389/fpsyt.2021.713279

**Published:** 2021-08-11

**Authors:** Yawen Guo, Haibo Yang, Jon Elhai, Dean McKay

**Affiliations:** ^1^Faculty of Psychology, Tianjin Normal University, Tianjin, China; ^2^Department of Psychology, University of Toledo, Toledo, OH, United States; ^3^Department of Psychology, Fordham University, New York, NY, United States

**Keywords:** COVID-19, attentional control, anxiety, anxiety sensitivity, cognitive-affective factor

## Abstract

**Background:** As an emergent public health event, COVID-19 has had a significant impact on mental health, particularly causing anxiety. Some cognitive-affective related studies have demonstrated that attentional control is related to levels of anxiety. More specifically, recent research has shown that anxiety sensitivity is uniquely associated with mental health responses to COVID-19. The aim of the current study was to investigate the role of anxiety sensitivity during COVID-19 outbreak period, especially physical and cognitive concerns, in relation to attentional control and anxiety.

**Methods:** It is a questionnaire study. A total of 464 participants were recruited through online sampling between February and March, 2020. They were surveyed by the Attentional Control Scale (ATTC), Anxiety Sensitivity Index-3 (ASI-3) and Depression Anxiety Stress Scale-21 (DASS-21). Data were analyzed using descriptive statistics and correlation analysis. We also tested the mediating effect.

**Results:** The results showed that attentional control is negatively correlated with physical concern, cognitive concern and anxiety. And results support that physical and cognitive concerns play a mediating role between attentional control and anxiety.

**Conclusions:** Anxiety sensitivity plays a mediating role between attentional control and anxiety. These findings can help effective prevention and intervention of anxiety.

## Introduction

The coronavirus disease 2019 (COVID-19) has become a huge threat all over the world as a pandemic, many countries are currently experiencing the second or third wave of the on-going COVID-19 pandemic, which may also lead to mental health problems of different groups. Recently, there have been numerous studies on individual anxiety during the pandemic (1–3). Wang et al. (3) found that the most common emotional response of people during the pandemic is anxiety. And another study found that prevalence of anxiety among college students during this pandemic was 27% (4). However, less is known about psychological factors that contribute to anxiety during the pandemic.

Anxiety is an aversive emotional and motivational state occurring in threatening circumstances (5). A large number of studies have shown that anxiety has important effects on physical and mental health (6), academic performance (7, 8), and interpersonal relationships (9). In the context of a widespread threat of infection, anxiety is expected, but since larger proportions of the population experience it, the consequences are more evident. Individuals with pre-existing anxiety conditions, has more severe consequences (10).

Previous studies showed that cognition and emotion are interdependent and interactive, and their neural mechanisms have functional integration, which together constitute the basis of behavior (11–13). Cognition is a necessary condition for emotion. As a part of cognitive processing, attention impacts emotional experience. For example, previous work found that in a preview search task, previewed distractors were consistently devalued, as compared with non-previewed distractors, despite longer exposure and being associated with an easier task (14). Attention bias is a phenomenon whereby individuals have higher sensitivity, and pay selective attention to specific stimuli (15). From an evolutionary perspective, this bias has adaptive meaning. Attention bias not only affects individuals' behaviors, but also their emotions (16). Attention bias to emotional information is an important factor in maintaining people's anxiety (17).

Attentional control is the general ability to regulate attention related to positive and negative reactions, reflecting the voluntary control of attention guided by expectations and motivations (18). Attentional control plays an important role in regulating the response to threat information. Many studies have explored the relationship between attentional control and negative emotions, such as anxiety symptoms (5, 19), and depression symptoms (20, 21). These studies showed that attentional control is an important contributor to anxiety and depression. Reinholdt-Dunne et al. (21) further evaluated the relationship between the two dimensions of attentional control and anxiety and depression. They found that a higher ability to concentrate is associated with a lower level of anxiety, and a higher ability to shift attention is associated with a lower level of depression.

Studies on anxious individuals have found that impaired attentional control systems can make individuals more likely to attend to threatening stimuli, which ultimately leads to increased anxiety levels (22). Derryberry and Reed (18) found that individuals with poor attentional control showed a bias toward threat stimuli after 500 ms, while individuals with better attentional control handled threat stimuli well and better diverted attention from threats. These studies suggest that attentional control affects anxiety by influencing a bias toward threatening stimuli. However, previous studies explored the direct effect of attentional control on anxiety, while lacking further investigation of the mediating variables between attentional control and anxiety. Exploring such mediating variables can help build up effective prevention and intervention of anxiety. The present study introduces another variable, anxiety sensitivity, to investigate the indirect effects of attentional control on anxiety.

Anxiety sensitivity refers to a cognitive-affective individual difference factor of the fear of bodily sensations, fearing that these sensations have harmful consequences (23). Individuals with high anxiety sensitivity tended to experience various negative emotions. It is a tendency to perceive symptoms of anxiety as being harmful, has been postulated as a cognitive risk factor for the development of anxiety disorders (24). Since the formulation of the anxiety sensitivity construct, it has been shown associated with the full range of anxiety disorders (25–28). As a part of cognitive processing, attentional control is expected to affect the emotional response, and thus affect anxiety sensitivity. Most existing studies focused on anxiety sensitivity as a whole, but few studies have explored its specific dimensions. Anxiety sensitivity is comprised of three broad sub-components: physical concern, cognitive concern, and social concern. Physical concern refers to the fear of physical sensations caused by anxiety, cognitive concern refers to the fear of losing control of cognitive or mental incompetence when facing stress or anxiety, and social concern is the apprehension that others will observe the sufferer experiencing anxiety. In light of increased concerns over infection during the COVID-19 pandemic, it would be expected that the public have greater interoceptive awareness, in a manner similar to anxiety sensitivity. Recent analyses have suggested a prominent role for anxiety sensitivity in fear of contracting COVID (1).

**Figure 1:**
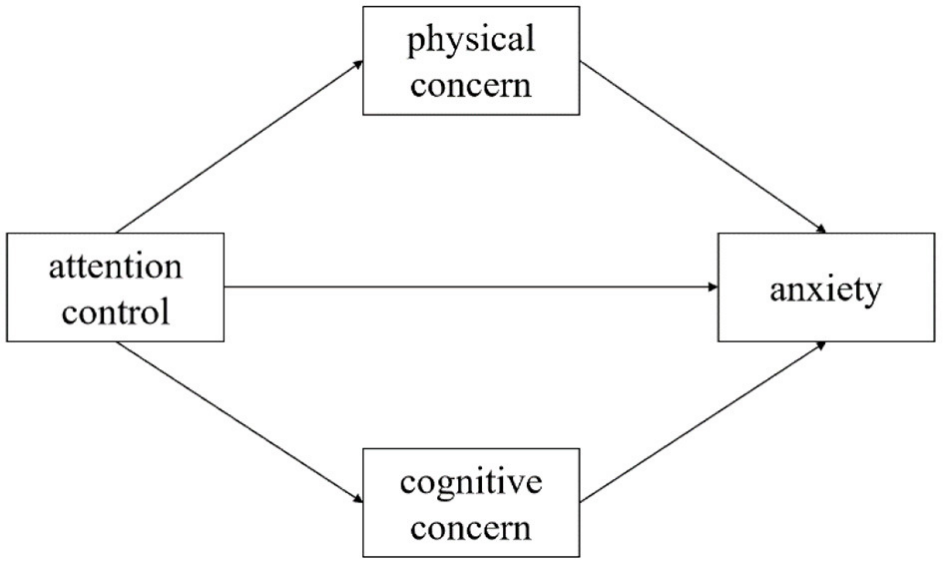
Mediation model tested.

In summary, it can be seen that there is a correlation between attentional control and anxiety, but this relationship may be regulated by anxiety sensitivity. Therefore, this study surveyed people during the COVID-19 pandemic to evaluate the role of anxiety sensitivity during this particular period, especially two dimensions, physical concern and cognitive concern, in relation to attentional control and anxiety. This study proposes the hypothesis (as shown in Figure 1): physical and cognitive concerns play mediating roles between attentional control and anxiety.

## Methods

### Participants and Procedure

We conducted a cross-sectional online survey of Chinese adults between February-March, 2020. Participants were invited by using the commonly-used Chinese social media app “WeChat” (29). Participants were primarily from Tianjin, but there were also respondents from 28 other provinces including Henan, Hebei, Sichuan, and Shanxi in China. Interested participants were shown an online informed consent statement and, for those agreeing, a Chinese language online survey hosted on Survey Star. All procedures performed in the present study were in accordance with the ethical standards of the Chinese Psychological Society (https://www.cpsbeijing.org/) and with the 1964 Helsinki Declaration and its later amendments or comparable ethical standards. The study was approved by the local ethics committee at Tianjin Normal University, Tianjin, China. Informed electronic consent was obtained from all participants included in the study.

A total of 569 people participated and completed the survey, after excluding those who answered too quickly, those whose answers had not been seriously considered (the answers are inconsistent or the answers are the same for at least ten consecutive questions), and those whose scores were outside ±3 standard deviations.

### Instruments

#### Attentional Control Scale

The ATTC is a 20-item self-report instrument that assesses general ability of participants in attentional control (18). Items are rated along a four-point scale from “1 = almost never” to “4 = always.” Higher scores indicate better attentional control. Internal consistency (coefficient alpha) for the present sample was 0.81.

#### Anxiety Sensitivity Index-3rd Edition

The ASI-3 is an 18-item measure that assesses anxiety sensitivity along three dimensions—physical, social, and cognitive concerns—with 6 items for each subscale (30). There is also a valid total score. Items are rated along a five-point scale from “0 = very little” to “4 = very much.” The Chinese version was employed in this study, which has a comparable factor structure (31). This study selected the physical concern and cognitive concern dimensions. Internal consistency for the present sample was 0.87 for physical concern and 0.86 for cognitive concern.

#### Depression Anxiety Stress Scale-21

The DASS-21 is a 21-item self-report instrument, with symptom ratings over the past week. The instrument employs a Likert-type scale from “0 = Did not apply to me at all” to “3 = Applied to me very much, or most of the time.” We analyzed only the 7 anxiety items. Lovibond and Lovibond (32) categorized DASS-21 anxiety scores into normal (0–7), mild (8–9), moderate (10–14), severe (15–19), and extremely (≥20). The anxiety subscale has adequate reliability and validity (33). We used the Chinese version, validated previously (34). Internal consistency in our sample was 0.82 for anxiety.

### Analysis

Analyses were conducted using the SPSS software package (v. 26.0 for Windows; IBM Corp., Armonk, NY, USA). Mediation tests (displayed in Figure 1) were conducted using the PROCESS macro (35). Descriptive statistics and correlation analysis were used. We used the bias-corrected non-parametric percentile Bootstrap confidence interval method to analyze the mediating effect.

## Results

### Descriptive Statistics

There was a final valid sample of *N* = 464 participants (75 males and 389 females), with an effective response rate of 81.55%. Mean age was 21.55 years (*SD* = 4.00), and most participants were women (*n* = 389, 83.84%). The distribution of anxiety levels on the DASS-21 in this study is shown in Table 1.

**Table 1:** The distribution of anxiety levels in this sample.

	**Anxiety level**				
	**Normal (*n* = 255)**	**Mild (*n* = 41)**	**Moderate (*n* = 94)**	**Severe (*n* = 34)**	**Extremely (*n* = 40)**
Female (%)	87.06	87.80	82.98	76.47	67.50
Age, *M* ±*SD*	21.28 ± 4.15	22.07 ± 5.18	21.88 ± 3.79	21.41 ± 2.87	22.08 ± 2.41
**Education level**
Middle school (%)	0	2.44	2.13	0	2.50
High school (%)	0.78	12.20	2.13	5.88	2.50
Secondary vocational technical school (%)	1.18	0	2.13	5.88	5.00
University (bachelor degree) (%)	84.31	68.29	82.98	73.53	67.50
University (MD/PhD) (%)	13.73	17.07	10.64	14.71	22.50

Table 2 displays the Pearson correlation matrix and descriptive information on the sample and measures used in the primary analyses. 464 participants had attentional control scores ranging from 31 to 75 (*M* = 52.108, *SD* = 7.134), anxiety scores from 0 to 28 (*M* = 7.647, *SD* = 6.950), physical concern scores from 0 to 21 (*M* = 6.810, *SD* = 5.061), and cognitive concern scores from 0 to 21 (*M* = 6.461, *SD* = 4.788).

**Table 2:** Correlation matrix of attentional control, physical concern, cognitive concern, and anxiety (*n* = 464).

**Variable**	***M* ±*SD***	**1**	**2**	**3**	**4**
1. Attentional control	52.108 ± 7.134				
2. Physical concern	6.810 ± 5.061	−0.329^**^			
3. Cognitive concern	6.461 ± 4.788	−0.410^**^	0.782^**^		
4. Anxiety	7.647 ± 6.950	−0.386^**^	0.551^**^	0.595^**^	

** *p < 0.01*.

There were significant correlations between the different variables. People with lower attentional control ability had a higher level of physical concern and cognitive concern, and showed greater anxiety.

### Mediation Models

**Figure 2:**
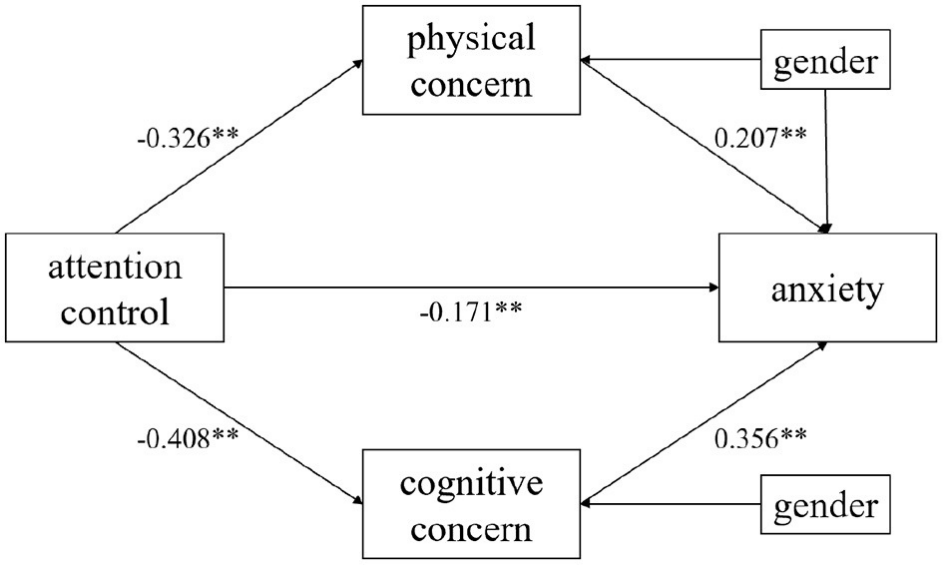
Multiple mediation model. **p < 0.01.

According to the hypothesis, the bias-checked non-parametric percentile confidence interval Bootstrap method was used to test for mediating effects. Due to the gender imbalance of participants, the gender factor was controlled. Five thousand Bootstrap samples were drawn to estimate 95% confidence intervals for mediating effects, and multiple mediation analyses were conducted with attentional control as the independent variable, physical and cognitive concerns as mediating variables, anxiety as the dependent variable, and gender as the covariate variable. The results are shown in Table 3, and the multiple mediation model obtained from the results is shown in Figure 2. The *p-*value of each path is lower than 0.01. From the model, attentional control not only directly predicted anxiety, but also indirectly predicted anxiety through physical and cognitive concerns.

**Table 3:** Regression analysis of relationships among model variables.

**Outcome variable**	**Predictor**	***R***	***R^**2**^***	***F***	***B***	***t***
Anxiety		0.410	0.168	46.536		
	Gender				−2.583	−3.223^**^
	Attentional control				−0.373	−9.019^**^
Physical concern		0.349	0.122	31.870		
	Gender				−1.590	−2.651^**^
	Attentional control				−0.231	−7.470^**^
Cognitive concern		0.418	0.175	48.909		
	Gender				−1.097	−1.996^*^
	Attentional control				−0.274	−9.640^**^
Anxiety		0.636	0.404	77.734		
	Gender				−1.565	−2.284^*^
	Physical concern				0.284	3.562^**^
	Cognitive concern				0.517	5.955^**^
	Attentional control				−0.166	−4.317^**^

* *p < 0.05,*

** *p < 0.01*.

The total effect size of the model was −0.373 and the direct effect size was −0.166. Physical and cognitive concerns partially mediated the relationship between attentional control and anxiety. The 95% confidence interval for the indirect effect of “attentional control → physical concern → anxiety” was (−0.115, −0.022), with a mediated effect size of−0.066. The 95% confidence interval for the indirect effect of “attentional control → cognitive concern → anxiety” was (−0.209, −0.086), with a mediated effect size of−0.142. The total indirect effect size was −0.207 (Table 4). The 95% confidence interval of each path did not include 0, indicating that the mediating effects of physical and cognitive concerns were statistically significant. These findings further support the hypothesis that physical concern and cognitive concern have significant mediating effects between attentional control and anxiety.

**Table 4:** Bootstrap analysis of mediation effect significance test.

	**Effect**	**BootSE**	**95% confidence interval**	
			**Upper limit**	**Lower limit**
M1 physical concern	−0.066	0.024	−0.115	−0.022
M2 cognitive concern	−0.142	0.032	−0.209	−0.086
M total	−0.207	0.029	−0.268	−0.154

## Discussion

At present, increasing empirical research has examined the impact of anxiety-related cognitive risk factors on healthy people. We proposed the present study based on the research idea of previous studies related to COVID-19 (36–38), exposing the potential mechanisms of attentional control on anxiety through path analysis. The purpose of this study was to investigate the role of physical and cognitive concerns, in relation to attentional control and anxiety. Our results provide support for the hypothesis that regarding the positive effects of attentional control during the pandemic: Attentional control is strongly associated with anxiety and anxiety sensitivity. There was a significant negative correlation between better attentional control ability and anxiety, and this negative connection can be explained by physical and cognitive concern dimensions of anxiety sensitivity.

### The Relationship Between Attentional Control and Anxiety

This study examined the relationship between attentional control and anxiety during the pandemic, and results build on prior research. High attentional control has been found to influence individuals' attention bias, making them less sensitive to threatening stimuli when confronted with negative information, as well as allowing the individual to shift attention better away from threat stimuli (18, 39), which is supported by this study. During the pandemic, individuals with higher attentional control have a better ability to focus and divert attention from distressing stimuli, including interoceptive experiences putatively associated with threat. On the one hand, they do not pay excessive attention to negative information associated with the pandemic; on the other hand, even if they do, they can immediately divert attention away from this information. Therefore, the emotional response caused by this information is relatively weak, and anxiety levels are relatively low. At the same time, results also found that individuals with higher attentional control ability have lower levels of anxiety sensitivity.

### Physical Concern and Anxiety

Bardeen and Daniel (40) found that anxiety sensitivity has a predictive effect on anxiety, which is supported by this study. This study found that individuals had higher levels of anxiety, partly due to greater fear of anxiety-related sensations, and this fear partly comes from fear of physical sensations caused by anxiety. In terms of physical concern, anxiety sensitivity has the characteristic tendency to further amplify the anxiety response (41). Studies from prior virus outbreaks found that physical concern scores were associated with fear of contracting illness (42, 43). McKay et al. (1) found that physical concern can be a predictor of fear of contracting COVID-19. During the pandemic, individuals have been extremely sensitive to their own health conditions. They may have some physical symptoms due to anxiety, such as rapid heartbeat or chest tightness, and respond to minute changes in physical sensations as indicative of threat (in this case, infection). The findings from this study suggest, however, that attentional control may influence the fear-evoking experience of awareness of interoceptive changes during the pandemic.

### Cognitive Concern and Anxiety

This study also found that the fear of anxiety-related feelings also partly comes from the fear of losing control of cognitive or mental functioning. Previous studies found that cognitive concern is related to anxiety (44), which is supported by this study. At the same time, Fergus et al. found that when there are mild physical symptoms, the correlation between anxiety and cognitive concern is stronger. A common concern among the public during the COVID-19 pandemic has been the extent that their cognitive functioning will remain intact [i.e., due to loss of social capital; (10)], and thus cognitive concern associated with anxiety sensitivity is highly salient. This finding is an extension from the original findings on the benefits of attentional control in managing anxiety reactions (18).

During the COVID-19 pandemic, people often have had mild physical symptoms such as rapid heartbeats due to anxiety, which makes a strong correlation between cognitive concern and anxiety. There are several aspects to the current understanding of cognitive concern. Some researchers believe that cognitive concern is related to fear of cognitive dissonance (30), while others believe that cognitive concern is highly correlated with repetitive thinking (45). Whereas Wheaton et al. (46) argued that cognitive concern in anxiety sensitivity is equivalent to negative meta-cognitive beliefs, which are associated with the belief that thoughts are dangerous (e.g., “I have lost control of my thoughts”) or threatening (e.g., “I may lose my mind because of worry”) (47), negative meta-cognitive beliefs were found to make anxiety difficult to regulate, thereby exacerbating feelings of threat (48), which in turn increases anxiety levels. The findings from this study deserve additional research, particularly in identifying methods of improving attentional control with corresponding examination of changes in anxiety sensitivity and fear of contracting COVID-19 or other infectious diseases. Recent research has shown that smartphone-based attentional control training has been effective in alleviating anxiety (49). Rapid administered interventions of this sort could be implemented to further isolate the effects of attentional control on anxiety disorder processes.

**Figure 3:**
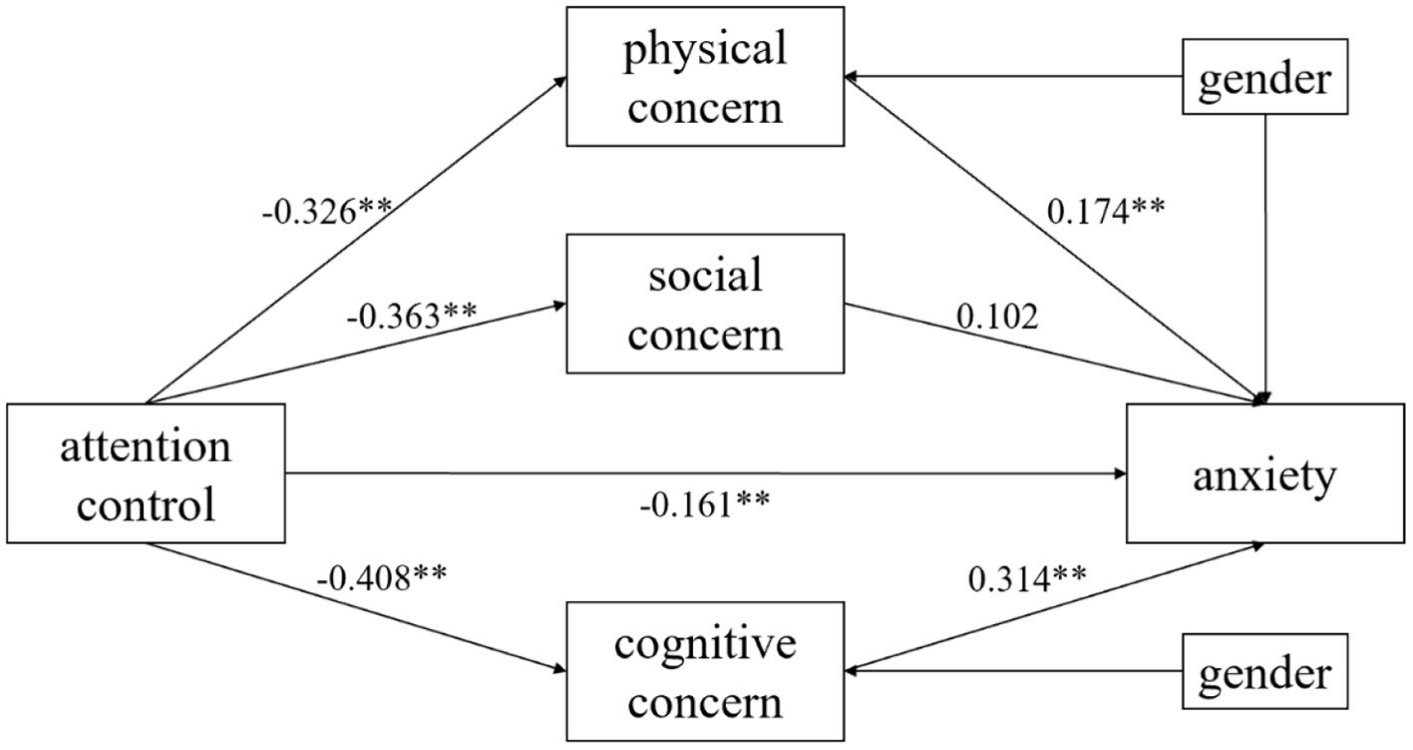
Multiple mediation model (physical, social, and cognitive concerns as mediating variables). **p < 0.01.

In addition, this study had used three dimensions of anxiety sensitivity—physical concern, cognitive concern, and social concern as mediating variables for further analysis, and the results showed that the regression coefficient of social concern was not significant. And the 95% confidence interval for the indirect effect of “attentional control → social concern → anxiety” was (−0.072, 0.001)include 0. These showed the mediating effect of social concern was not significant (see Tables 5, 6; Figure 3), indicating that attentional control cannot affect anxiety levels through social concern. This suggests that during the pandemic, individuals' attention was primarily focused on his own situation, ignoring the external society's evaluation of himself, which has implications for future interventions when emergencies occur.

**Table 5:** Regression analysis of relationships among model variables (physical, social and cognitive concerns as mediating variables).

**Outcome variable**	**Predictor**	***R***	***R^**2**^***	***F***	***B***	***t***
Anxiety		0.410	0.168	46.536		
	Gender				−2.583	−3.223^**^
	Attentional control				−0.373	−9.019^**^
Physical concern		0.349	0.122	31.870		
	Gender				−1.590	−2.651^**^
	Attentional control				−0.231	−7.470^**^
Social concern		0.371	0.137	36.695		
	Gender				−1.004	−1.608
	Attentional control				−0.270	−8.377^**^
Cognitive concern		0.418	0.175	48.909		
	Gender				−1.097	−1.996^*^
	Attentional control				−0.274	−9.640^**^
Anxiety		0.639	0.409	63.317		
	Gender				−1.569	−2.297^*^
	Physical concern				0.239	2.890^**^
	Social concern				0.134	1.942
	Cognitive concern				0.456	4.944^**^
	Attentional control				−0.157	−4.067^**^

* *p < 0.05,*

** *p < 0.01*.

**Table 6:** Bootstrap analysis of mediation effect significance test (physical, social, and cognitive concerns as mediating variables).

	**Effect**	**BootSE**	**95% confidence interval**	
			**Upper limit**	**Lower limit**
M1 physical concern	−0.055	0.023	−0.104	−0.013
M2 social concern	−0.036	0.018	−0.072	0.001
M3 cognitive concern	−0.125	0.033	−0.195	−0.066
M total	−0.216	0.028	−0.276	−0.164

## Limitations

The strengths of this study are that the survey was conducted online, with a high number of participants (464), and using three scales with good reliability. The results can help effective prevention and intervention of anxiety.

This study still has some limitations. First of all, this study is a cross-sectional study, unable to investigate the direction between variables. Therefore it is not possible to know whether attentional control contributes directly or indirectly to the reduction of anxiety. Future studies can use longitudinal research or experimental methods to explore the causal relationship between variables. Secondly, the data in this research are all from self-reports. Objectivity and authenticity of data cannot be guaranteed. Future research should integrate other information channels to collect data, such as from family and friends. Data from different sources can be mutually confirmed, so that the research can obtain more objective results.

In sum, the current study indicates that attentional control is negatively correlated with physical concern, cognitive concern and anxiety, and physical and cognitive concerns play a mediating role between attentional control and anxiety.

## Data Availability Statement

The raw data supporting the conclusions of this article will be made available by the authors, without undue reservation.

## Ethics Statement

The studies involving human participants were reviewed and approved by The Ethics Committee of Tianjin Normal University. Written informed consent for participation was not required for this study in accordance with the national legislation and the institutional requirements.

## Author Contributions

HY designed the study protocol. YG conducted data collection and conducted data management, cleaning, and analysis. YG and HY wrote the first draft of the paper. JE and DM substantially revised the manuscript. All authors contributed to the article and approved the submitted version.

## Conflict of Interest

The authors declare that the research was conducted in the absence of any commercial or financial relationships that could be construed as a potential conflict of interest.

## Publisher's Note

All claims expressed in this article are solely those of the authors and do not necessarily represent those of their affiliated organizations, or those of the publisher, the editors and the reviewers. Any product that may be evaluated in this article, or claim that may be made by its manufacturer, is not guaranteed or endorsed by the publisher.
